# The predictive value and clinical application of measuring circulating endothelial progenitor cells in lung adenocarcinoma

**DOI:** 10.1186/s13019-026-04144-3

**Published:** 2026-04-21

**Authors:** Lei Zhang, Zongfei Liu, Yuehua Zhang, Zheng Wu, Ya Liu, Zhiyu Wang

**Affiliations:** 1 Department of Immuno-Oncology, The Fourth Hospital of Hebei Medical University, No. 12 Jiankang Road, Shijiazhuang, 050011 Hebei China; 2 Department of Oncology, The Second People’s Hospital of Hengshui, Hengshui, 053099 Hebei China

**Keywords:** Endothelial Progenitor Cell, Lung Adenocarcinoma, Neovascularization, VEGF, Prognosis

## Abstract

**Background:**

This study investigated the potential of endothelial progenitor cells (EPCs) as biomarkers for lung adenocarcinoma.

**Methods:**

Blood samples were collected from 34 lung adenocarcinoma patients and 8 healthy controls at the Department of Tumor Immunology and Oncology. EPCs were quantified using flow cytometry, while serum VEGF levels were measured via enzyme-linked immunosorbent assays (ELISA). Comparative analyses between groups were performed, and correlations between EPC counts and VEGF levels were assessed. Bioinformatics analysis further explored VEGF expression in lung adenocarcinoma and its relationship with patient survival.

**Results:**

Peripheral blood EPC counts were significantly elevated (7.36-fold higher) in treatment-naïve patients (*n* = 12) compared to healthy controls (*n* = 8) (*p* < 0.05). The chemotherapy group (*n* = 11) showed a further 1.05-fold increase compared to the treatment-naïve group (*p* < 0.05), while the chemotherapy+bevacizumab group (*n* = 11) demonstrated a 0.65-fold reduction compared to the chemotherapy group (*p* < 0.05). Serum VEGFA and VEGFB levels were significantly higher in patients than controls (0.08-fold and 0.11-fold increases, respectively; both *p* < 0.05). Positive correlations were observed between serum VEGFA/VEGFB levels and peripheral blood EPC counts (*r* = 0.593, *p* = 0.001; *r* = 0.440, *p* = 0.009, respectively). Bioinformatics analysis confirmed elevated VEGFA and VEGFB mRNA expression in lung adenocarcinoma patients compared to healthy individuals.

**Conclusion:**

Circulating EPCs may serve as valuable biomarkers for lung adenocarcinoma diagnosis and prognosis. These findings provide a theoretical foundation for incorporating EPC assessment in early lung cancer screening and monitoring targeted therapy effectiveness in lung adenocarcinoma patients.

## Introduction

Lung cancer represents the leading cause of cancer-related mortality worldwide, with an increasing incidence trend in China [1, 2]. Currently, adenocarcinoma accounts for approximately 40% of all lung cancers, which is the most prevalent type of lung cancer among non-smokers [3]. With ongoing advancements in medical technology, treatment approaches for lung cancer have become increasingly diversified. Beyond traditional surgery, radiotherapy, and chemotherapy, various therapies including targeted therapy and immunotherapy continue to update clinical guidelines, substantially improving survival duration and quality of life for patients with advanced lung cancer [4]. However, survival rates are still low due to late diagnoses, with 40% of patients diagnosed only after malignant tumors have metastasized beyond the lungs [5]. This underscores the necessity of establishing effective screening methods to identify lung cancer at early stages [6].

Biomarker identification offers another effective option for lung cancer screening. This method primarily identifies molecules, such as cytokines, that circulate in the blood due to active processes associated with lung cancer [7]. Vascular endothelial growth factor (VEGF), a multifunctional cytokine, is widely expressed in various cancer cells. In non-small cell lung cancer, VEGF serves as the primary mediator promoting tumor microvasculature formation and is closely associated with disease progression, recurrence, and metastasis [8, 9]. These anti-tumor angiogenesis therapies not only promote normalization of abnormal tumor vasculature but also influence immune responses by modulating the VEGF/VEGFR signaling pathway [10–12].

Endothelial progenitor cells (EPCs), involving the formation of new blood vessels, plays a crucial role in tumor growth and metastasis [13–15]. In clinical practice, numerous studies have demonstrated that EPCs play important roles in the diagnosis, treatment, and prognosis of malignant tumors such as gastric cancer [16], gynecological tumors [17], and breast cancer [18]. Research by Maeda et al. revealed differences in circulating EPC numbers and intratumoral microvessel density among different lung adenocarcinoma histologic subtypes, reflecting varying angiogenic states [19]. Recently, Najjar et al. confirmed the significant value of endothelial progenitor cells as an angiogenic biomarker for the diagnosis and prognosis of lung cancer [20]. However, few studies have investigated circulating EPCs in adenocarcinoma, leaving uncertainty about their potential clinical value as biomarkers for screening or prognosis during diagnosis.

This study aims to explore whether EPCs can serve as biomarkers for predicting prognosis and anti-angiogenic treatment efficacy in lung adenocarcinoma. We analyzed EPCs in patients using flow cytometry and correlated VEGF levels using enzyme-linked immunosorbent assay (ELISA). The bioinformatic analysis was used to explore potential associations between VEGF levels and lung adenocarcinoma and patient survival. We present this article according to the STROBE reporting checklist.

## Materials and methods

### Patient inclusion and study grouping

From January 2019 to June 2019, 34 patients with advanced lung adenocarcinoma were enrolled from the Department of Tumor Immunology and Oncology, Fourth Hospital of Hebei Medical University. All enrolled patients were pathologically diagnosed with lung adenocarcinoma and had not received any other anti-tumor treatments such as radiotherapy, interventional therapy, targeted drugs, or immunotherapy.

After diagnosis, the patients received treatment according to the National Comprehensive Cancer Network and the Chinese Society of Clinical Oncology guidelines. Doctors recommend treatment plans tailored to each patient’s specific situation and provide detailed explanations for each option. The patients and their families discussed and chose the treatment plan that best fit their needs. The treatment involved phase IV systemic drugs, pemetrexed combined with cisplatin chemotherapy, with or without targeted angiogenesis therapy with bevacizumab. The patient voluntarily participated in this study.

Eight healthy individuals were selected as the control group to maintain broad parity between the individual groups. The 34 enrolled patients were grouped according to their first-line treatment. Therefore, this study included a healthy-controls group (*n* = 8), a treatment-naïve group (*n* = 12; no anti-tumor treatment given), a chemotherapy group (*n* = 11; treated with monotherapy of pemetrexed or pemetrexed plus cisplatin), and a chemotherapy + bevacizumab group (*n* = 11; treated with bevacizumab in addition to chemotherapy).

### Sample collection

Each patient in the study provided two milliliters of fasting peripheral blood for analysis at the time when they were pathologically diagnosed with lung adenocarcinoma and had not received any treatment. The samples of fasting peripheral blood in the control group were also collected. The samples from treatment-naïve group were detected and only used for reference and study in this current study. In the clinical practice, after the samples collection, treatment-naïve group received treatment. For the patients in the chemotherapy and chemotherapy + bevacizumab groups, fasting peripheral blood was collected again at the end (the 21st day) of the first cycle of treatment.

### Flow cytometry

Ten microliters of CD45 monoclonal antibody, 10 µl of VEGFR-2 monoclonal antibody, 3 µl of CD133 monoclonal antibody, and 3 µl of CD34 monoclonal antibody were added to a pre-prepared Eppendorf tube. The peripheral blood sample was slightly shaken to mix well, then 200 µl of blood was added to the corresponding Eppendorf tube and mixed well. After incubation at room temperature in the dark for 30 min, 3 ml of red blood cell lysis buffer was added and mixed well. After another 30 min at room temperature and away from light, it was centrifuged in a low-speed centrifuge (1200 r/min, 6 min). After centrifugation, the supernatant was discarded, then 2 ml phosphate buffered saline (PBS) was added, the tube was slightly shaken to make the mixture uniform, and then centrifuged in a low-speed centrifuge (1200 r/min, 6 min). The supernatant was discarded again, and then 200 µl PBS was added to ensure it was evenly mixed by slight shaking. The flow cytometry analysis was then carried out on the flow cytometer machine (FACS 3K976, B&D, USA) according to standard procedures.

### Enzyme-Linked Immunosorbent Assay (ELISA)

Detection of VEGFA and VEGFB in the serum samples was undertaken using ELISA kits from Abcam (UK). For the samples to be analyzed 40 µl of sample diluent and 10 µl of the tested sample were added to each well of the plate to achieve a final dilution of the sample by 5. Alongside this 50 µl of standards was added to the standard wells of the ELISA plate and some wells remained blank. The ELISA was then performed according to the instructions provided with the kit.

### Main reagents and instruments

CD-34 monoclonal antibody to detect human antigens, mouse anti-human APC-CD133, mouse anti-human PE-VEGFR-2, and mouse anti-human FITC-CD-45 monoclonal antibody were all purchased from BD Biosciences (USA); VEGFA and VEGFB ELISA kits were purchased from Abcam (UK); flow cytometer (BD, USA); low-speed centrifuge (Beijing Baiyang Medical Equipment Co., Ltd.); ELISA reader (Bio-Rad, USA); single-channel pipette (Shandong Bokang Scientific Instrument Co., Ltd.).

### Bioinformatics analysis

The Oncomine database (https://www.oncomine.com) was used to predict the difference in VEGFA and VEGFB mRNA expression between lung adenocarcinoma patients and healthy individuals. The online Kaplan-Meier website (https://kmplot.com) was used to analyze the relationship between VEGFA and VEGFB expression levels and the prognosis of lung adenocarcinoma patients.

### Statistical analysis

SPSS 21.0 statistical software (IBM Corp., USA) was used for statistical analysis. The count data used in the experiment were expressed as mean ± standard deviation (sd). Chi-square test was used for comparison between the healthy-controls group, treatment-naïve group, chemotherapy group, and chemotherapy + bevacizumab group. An independent sample t test was used to compare the VEGFA and VEGFB levels between healthy controls and patients with lung adenocarcinoma. Spearman’s rank correlation test was used for correlation analysis between VEGFA/VEGFB levels and peripheral blood EPCs in the serum of patients with lung adenocarcinoma. A *P* < 0.05 was considered statistically significant.

## Results

### Baseline characteristics

There were no significant statistical differences between the groups in the general information of the enrolled members, including age, gender, and previous history (diabetes, hypertension, smoking) (*P* > 0.05, Table 1). There were also no significant differences in differentiation degree of the tumors, stage of the tumor, duration of illness, or terms of chemotherapy cycles between the three adenocarcinoma groups, or between the two treatment groups (*P* > 0.05, Table 1).

**Table 1:** General information of group members

Variables	Healthy-	Patients with lung adenocarcinoma				*p*-value
	controls group (*n* = 8)	Treatment-naïve group (*n* = 12)	Chemotherapy group (*n* = 11)	Chemotherapy + bevacizumab group (*n* = 11)	χ^2^	
Age						
≤ 60	7	7	4	5	5.411	0.144
>60	1	5	7	6		
Gender						
Male	4	5	5	4	0.393	0.942
Female	4	7	6	7		
Differentiation degree of tumors						
Poorly differentiated		5	6	5	1.575	0.813
Moderately differentiated		6	3	5		
Highly differentiated		1	2	1		
Cycles of chemotherapy						
≤ 4			6	8	0.786	0.375
>4			5	3		
Stage of tumor						
Ⅲ		5	3	2	1.560	0.458
Ⅳ		7	8	9		
Duration of illness						
< 1year		11	8	11	4.151	0.125
1year		1	3	0		
Previous History						
Diabetes	2	2	0	1	3.137	0.371
Hypertension	4	4	2	3	2.299	0.513
Smoking	5	8	7	8	0.290	0.962

### Flow cytometry analysis

**Fig. 1:**
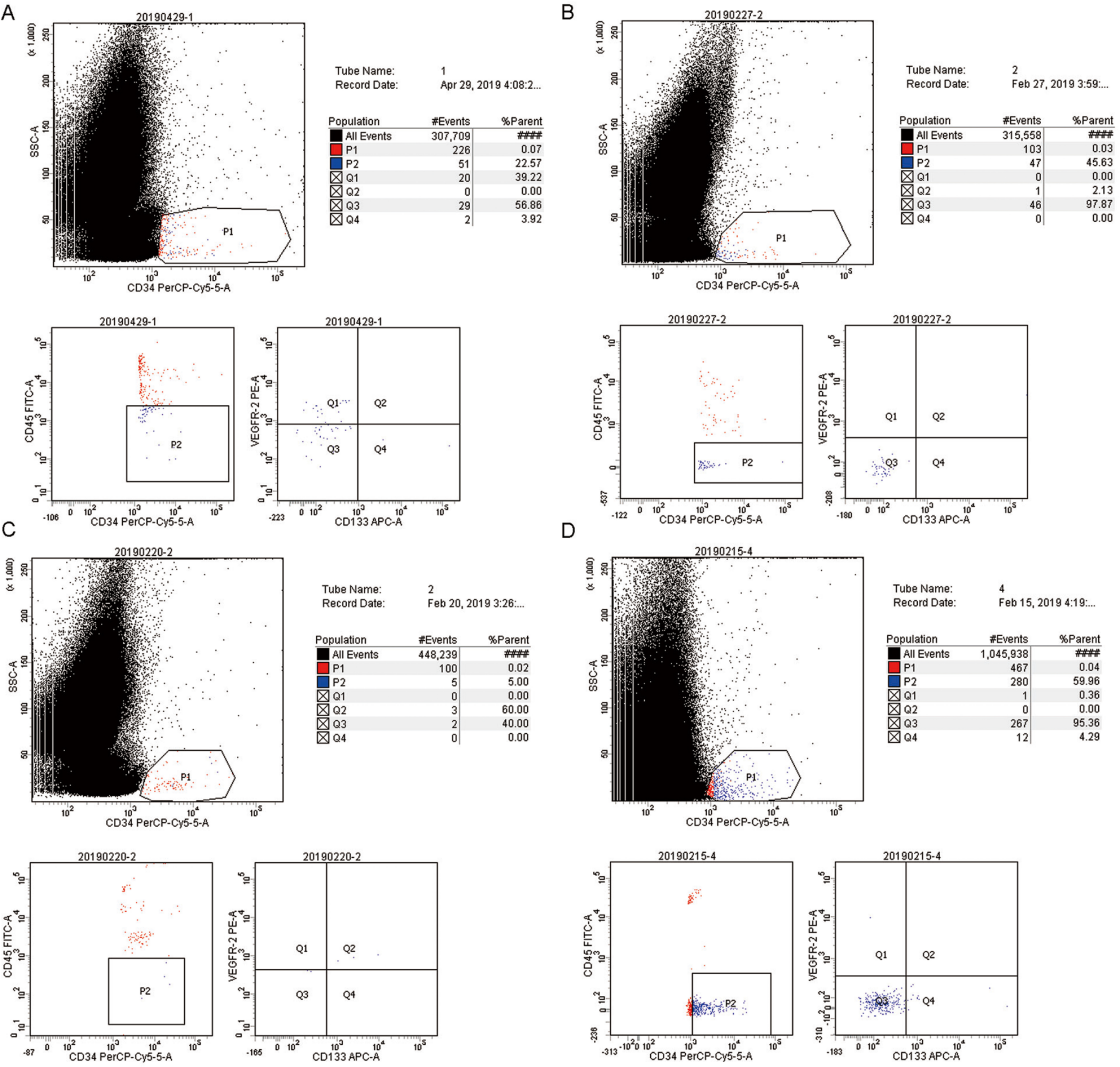
Flow cytometry analysis of EPCs in peripheral blood samples. (**A**) Expression of EPC in peripheral blood of the healthy-controls group. (**B**) Expression of EPC in peripheral blood of patients in the treatment-naïve group. (**C**) Expression of EPC in peripheral blood of patients in the chemotherapy group. (**D**) Expression of EPC in peripheral blood of patients in the chemotherapy + bevacizumab group

Flow cytometry was used to analyze and count EPC cells after staining with CD133 (AC133), CD34, VEGFR-2, and CD45 monoclonal antibodies as shown in Fig. 1.

**Fig. 2:**
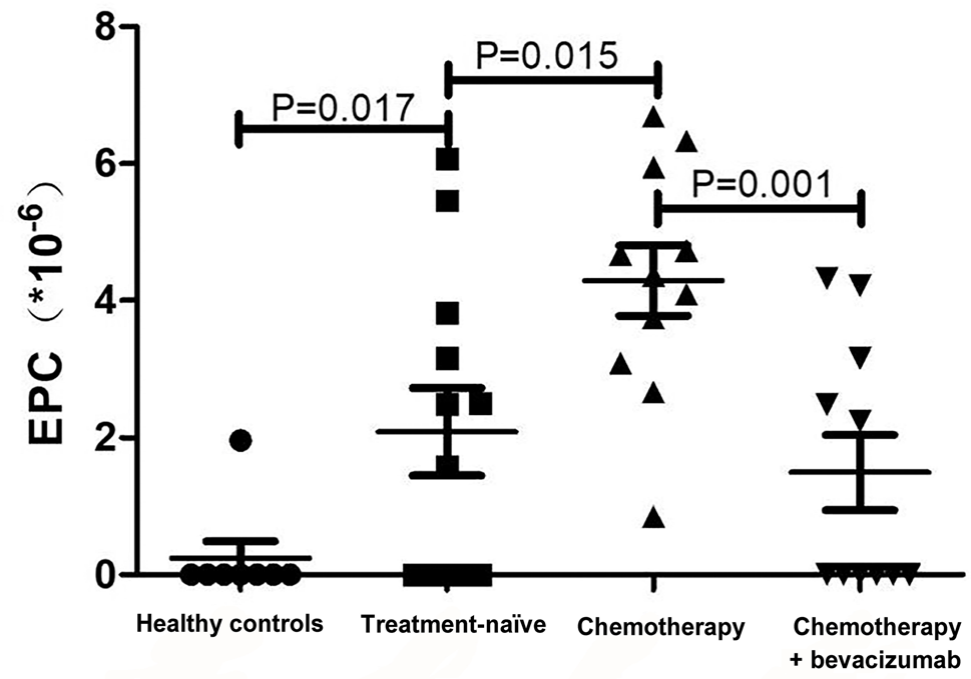
Comparison of EPC counts between the study groups. Significant statistical differences are indicated between the values (*P* < 0.05)

The EPC counts were 0.25 ± 0.648 in the healthy control group, 2.09 ± 2.123 in the treatment-naïve group, 4.29 ± 1.630 chemotherapy group, and 1.50 ± 1.736 in the chemotherapy + bevacizumab group. The EPCs were 7.36 fold higher in the treatment-naïve group than in the healthy control group, a significant difference (*P* = 0.017). The EPCs were 1.05 fold higher in the chemotherapy group than in the treatment-naïve group, a significant difference (*P* = 0.015; *P* < 0.05). The EPCs were − 0.65 fold lower in the chemotherapy + bevacizumab group than in the chemotherapy group, a significant difference (*P* = 0.001). The comparison of the EPC counts is shown in Fig. 2. These results suggest that EPC counts could identify patients with adenocarcinoma and were influenced by treatment.

### ELISA analysis

The ELISA analysis revealed that the levels of both VEGFA and VEGFB in the serum of the patients with lung adenocarcinoma were significantly higher than that in the control group (*P* = 0.001 and *P* = 0.004, respectively) as shown in Table 2. Therefore, this suggests both types of VEGF could be a biomarker for adenocarcinoma.

**Table 2:** Serum expression levels of VEGFA and VEGFB in both groups

Groups	VEGFA	VEGFB
Healthy controls (*n* = 8)	165.16 ± 7.94	176.18 ± 8.62
Patients with lung adenocarcinoma (*n* = 34)^#^	178.68 ± 10.18	194.81 ± 16.25
*P*	0.001*	0.004*

* *P* < 0.05 was statistically significant

^#^Patients with lung adenocarcinoma included the treatment-naïve group (*n* = 12), chemotherapy group (*n* = 11), and chemotherapy + bevacizumab group (*n* = 11)

### Correlation analysis

While this study showed that the serum VEGF of patients with adenocarcinoma of the lung was significantly higher than that of healthy people, it has previously been demonstrated that bevacizumab has an inhibitory effect on EPC mobilization in the peripheral blood. Bevacizumab is an anti-VEGF monoclonal antibody. Therefore, we performed correlation analysis between VEGFA and VEGFB levels in the serum of patients with lung adenocarcinoma and peripheral blood EPCs. There was a positive correlation between the expression level of VEGFA in the serum of lung adenocarcinoma patients and EPCs in peripheral blood (*r* = 0.593, *P* < 0.001); there was also a positive correlation between the expression level of VEGFB in the blood serum of lung adenocarcinoma patients and EPCs in peripheral blood (*r* = 0.440, *P* = 0.009; as shown in Table 3).

**Table 3:** Correlation analysis of serum VEGFA, VEGFB and peripheral blood EPCs in lung adenocarcinoma patients^#^

Index	Correlation coefficient (*r*)	*P* value
VEGFA (*n* = 34)	0.593	< 0.001*
VEGFB (*n* = 34)	0.440	0.009*

*P < 0.05 was statistically significant

^#^Patients with lung adenocarcinoma included the treatment-naïve group (*n* = 12), chemotherapy group (*n* = 11), and chemotherapy + bevacizumab group (*n* = 11)

### Bioinformatics analysis

**Fig. 3:**
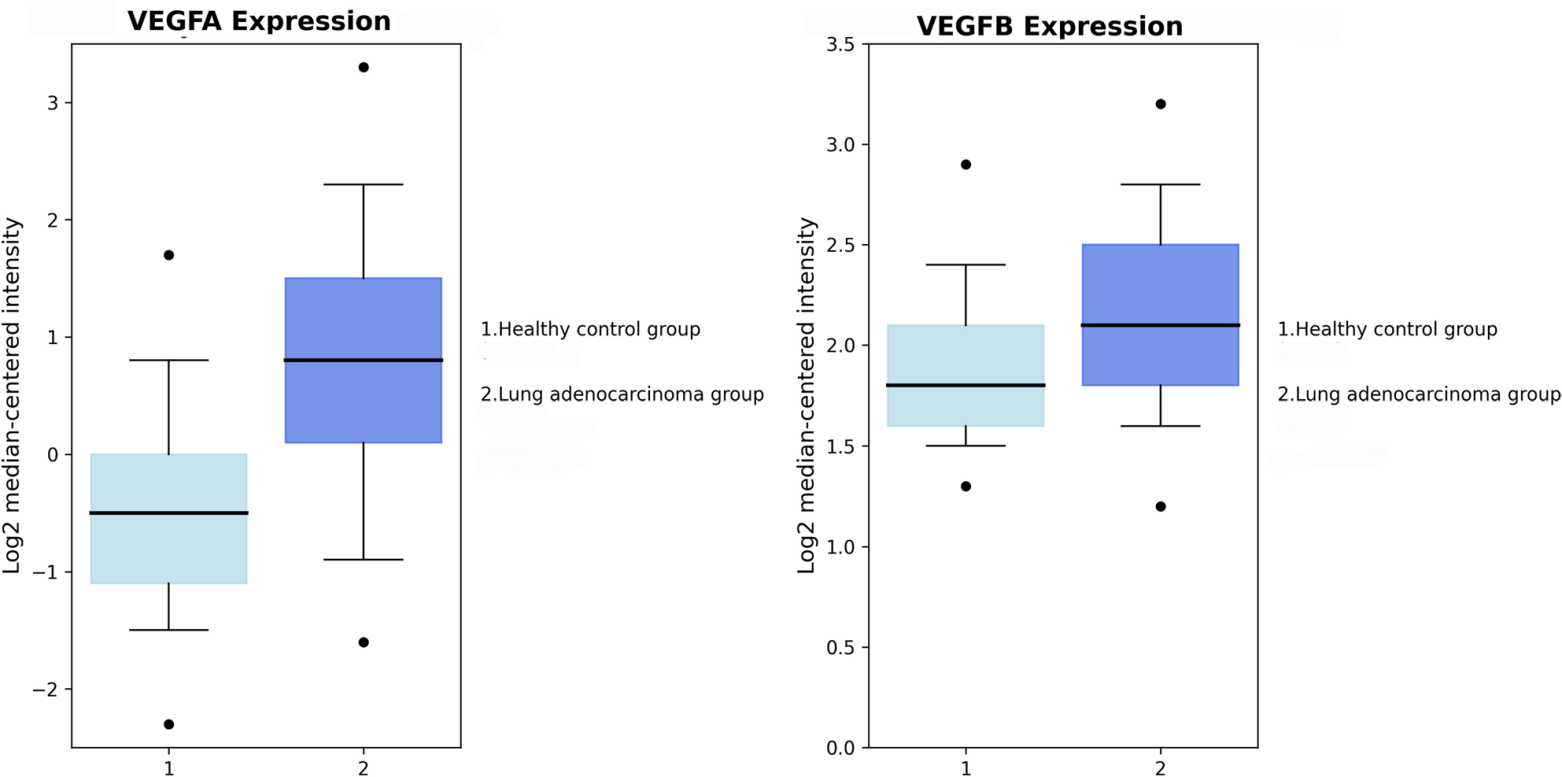
Differential analysis of VEGFA and VEGFB transgenes in the Oncomine database. Significant statistical differences are indicated between the values (*P* < 0.05)

To further investigate the association between VEGF and EPCs we next performed bioinformatics analysis. Using the Oncomine database, we verified that the expression levels of VEGFA and VEGFB are related to the expression level of EPCs in peripheral blood of patients with lung adenocarcinoma. The analysis showed that the expression levels of these genes were higher in lung adenocarcinoma patients than in normal individuals, with statistical significance (VEGFA: *P* = 9.36 × 10^− 8^; VEGFB: *P* = 5.50 × 10^− 4^; both P-value < 0.05; as shown in Fig. 3). This analysis supports the view that VEGF may be associated with EPCs in the patients with adenocarcinoma.

**Fig. 4:**
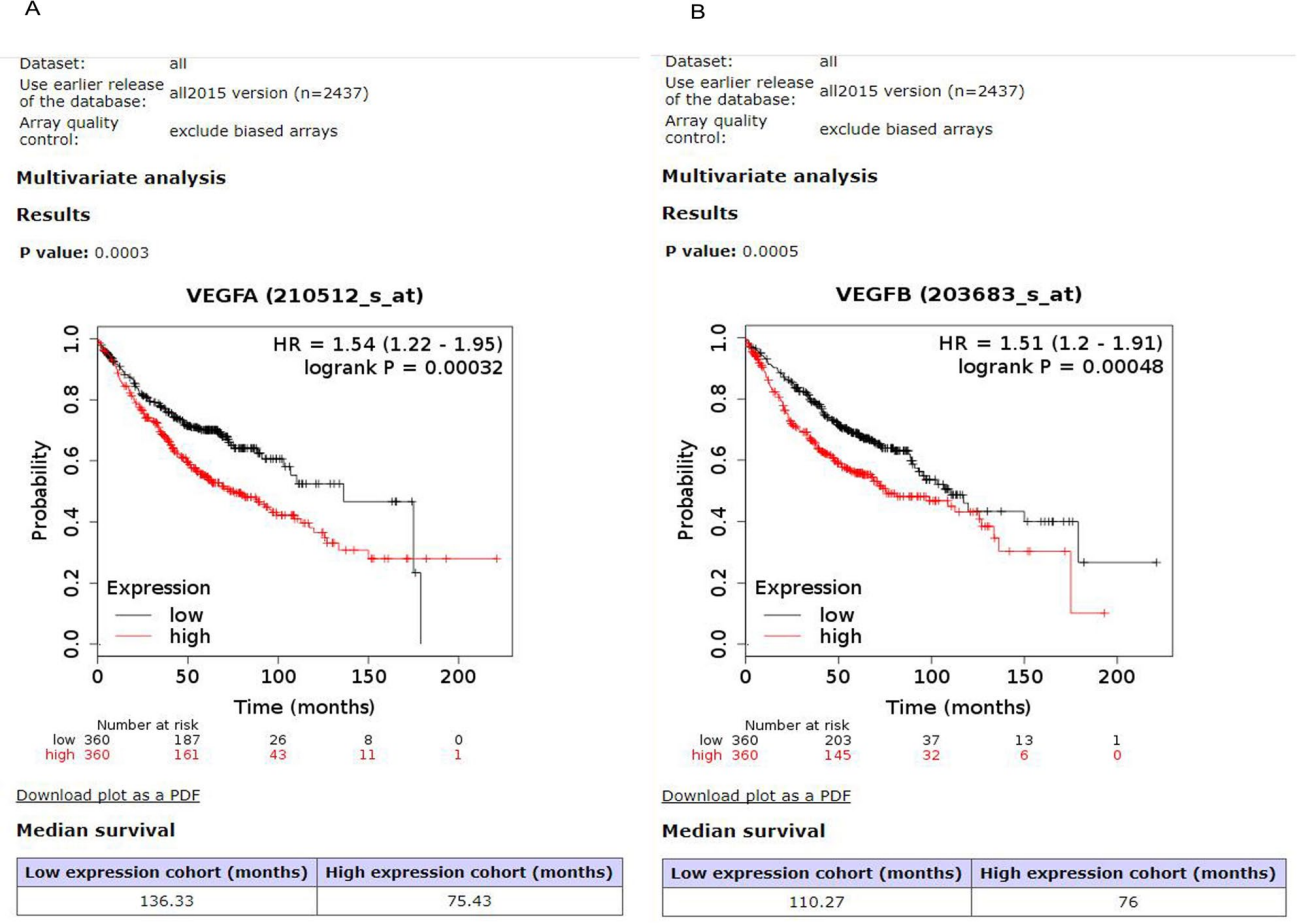
Kaplan-Meier survival curve analysis. (**A**) Survival curves of patients with low and high VEGFA levels; (**B**) Survival curves of patients with low and high VEGFB levels. Significant statistical differences are indicated between the survival curves (*P* < 0.05)

To establish a potential relationship with patient prognosis we next used the online Kaplan-Meier website to investigate a relationship between VEGF and survival rates. We found that the expression levels of VEGFA (median survival time in low-expression group: 136.33; median survival time in high-expression group: 75.43; *P* = 0.00032) and VEGFB (median survival time in low-expression group: 110.27; median survival time in high-expression group: 76; *P* = 0.00048) may have an inverse correlation with the prognosis of lung adenocarcinoma and have statistical significance (*P* < 0.05; as shown in Fig. 4). This result suggests that VEGF level could predict patient survival, and as VEGF is associated with EPCs then EPC counts may also be an indicator of patient survival.

## Discussion

Results in the current study showed that peripheral blood EPC levels were higher in untreated lung adenocarcinoma patients compared to the control group; EPC levels were even higher in patients receiving chemotherapy alone, while they decreased in patients receiving chemotherapy combined with bevacizumab. These results suggest that EPCs may serve as potential biomarkers for lung adenocarcinoma and its prognosis, which was validated through correlation analysis with VEGF levels and bioinformatics analysis - VEGF levels were found to be associated with the occurrence of lung adenocarcinoma and patient survival rates.

Our results indicate that in lung adenocarcinoma patients, EPCs are mobilized into peripheral blood, and treatment with pemetrexed alone or pemetrexed combined with cisplatin further increases the number of EPCs in peripheral blood. Similar phenomena have been reported in other types of cancer. Research reveals that EPC levels in blood are higher in liver, breast, and liver cancer patients, and lower EPC counts are linked to better survival rates in lung cancer patients [21–24]. Another study divided lung adenocarcinoma patients into high and low EPC groups, showing that in the high EPC group, patients receiving chemotherapy combined with bevacizumab had significantly better disease control rates and tumor reduction rates compared to those receiving chemotherapy alone; while in the low EPC group, there was no significant difference between the combined treatment group and the chemotherapy-only group [25]. In our study, EPC counts in the chemotherapy plus bevacizumab group were lower than in the chemotherapy-only group, which is different from the results by Sudo et al. [25].

Notably, Deng et al. revealed that crosstalk exists between EPCs and hepatocellular carcinoma, which promotes liver cancer metastasis through periostin/CCL2/CD36 signaling [26]. Campani et al. similarly found that in advanced hepatocellular carcinoma patients, EPCs were directly correlated with platelet count, and frequencies of all EPC populations declined in patients receiving sorafenib [27]. These findings are similar to our observations in lung adenocarcinoma, suggesting that EPCs may have common mechanisms of action in different types of cancer. However, it remains unclear whether the lower EPC counts in chemotherapy plus bevacizumab treatment indicate better prognosis, as bevacizumab is an anti-VEGF therapeutic agent that may in turn affect EPC mobilization. Doppelt-Flikshtain et al. showed that anti-VEGFA antibodies can reduce the effects of cytokines secreted by EPCs, thereby reducing tumor cell migration, which provides new insights into understanding the mechanism of bevacizumab [28].

VEGF helps move and multiply EPCs by binding to its receptor and activating downstream MAPK/ERK signaling pathways [29, 30]. Anti-angiogenic targeted drugs such as bevacizumab are commonly used in lung adenocarcinoma treatment, which may inhibit this phenomenon and potentially improve patient prognosis [31]. Previous studies have shown that high VEGF levels were associated with decreased survival rate of patients with lung adenocarcinoma and other types of lung cancer [32–35]. These results are also supported by our bioinformatics analysis: Kaplan-Meier analysis showed a significant relationship between high levels of VEGFA and VEGFB and shorter survival times.

## Limitations

This study has several limitations. The sample size was small because the subjects were drawn from a single hospital. Future inclusion of data from multiple centers would help enhance the credibility of the results. In addition, patient selection and treatment regimen formulation were based on clinical guidelines, potentially introducing some selection bias. When discussing relationships between systemic expression of VEGF and survival, the authors do not provide information of potential confounders or contributing factors. In addition, no further information is provided on other subsequent therapies, specifically on any targeted therapy or immune checkpoint inhibitors. Further research is needed to clarify the direct link between EPCs and patient prognosis with the control of contributing factors. Further information should be collected on other subsequent therapies, specifically on any targeted therapy or immune checkpoint inhibitors, to complete the whole investigation on these patients.

## Conclusion

In our study, VEGFA and VEGFB levels were positively correlated with peripheral blood EPC levels, indicating an interaction between EPCs and VEGF. This relationship may serve as an effective indicator for clinical assessment of disease status. EPC counts may associate with the existence of lung adenocarcinoma and may help on patient prognosis. Nevertheless, more research is needed to further validate this conclusion.

## Data Availability

The datasets used and/or analyzed during the current study are available from the corresponding author on reasonable request.

## Abbreviations

NSCLC: Non-small cell lung cancer
VEGF: Vascular endothelial growth factor
VEGFR: The VEGF and its receptor
EPCs: Endothelial progenitor cells
ELISA: Enzyme-linked immunosorbent assay
PBS: Phosphate buffered saline
